# Supplementary material for: CD200R1 Contributes to Successful Functional Reinnervation after a Sciatic Nerve Injury
Source: Cells. 2022 May 30;11(11):1786. doi: 10.3390/cells11111786 (PMC9179995; doi:10.3390/cells11111786)
Supplement: Supplementary file 1 [file cells-11-01786-s001.zip › cells-1680502-supplementary.pdf]

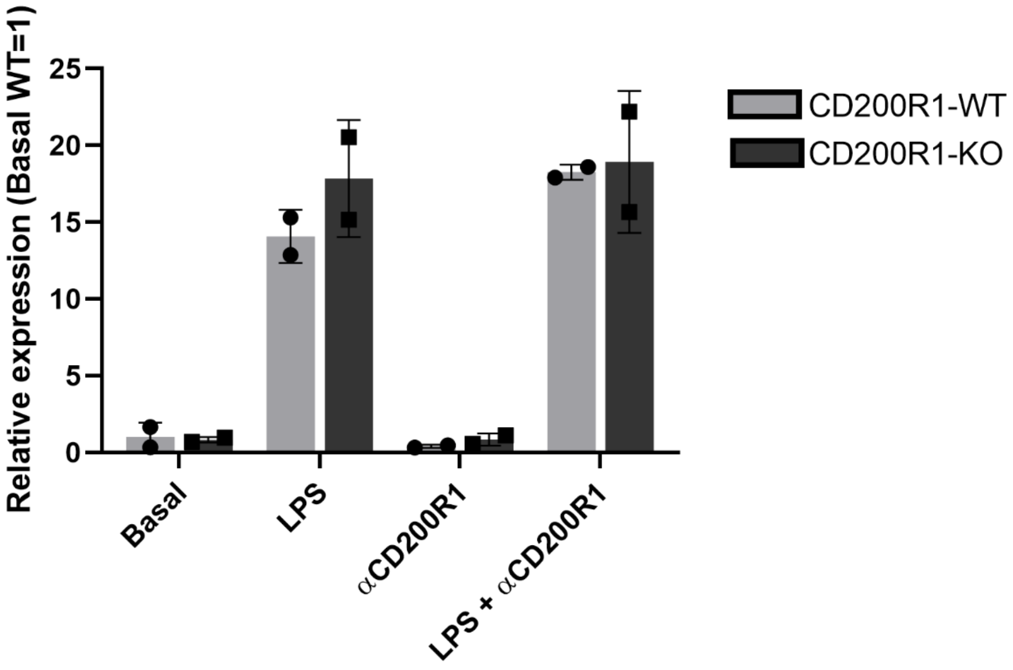

Supplementary Figure S1. αCD200R1 does not activate CD200R1 in WT or CD200R1-knockout derived BMDM. IL1b mRNA expression in BMDM from WT and CD2001-knockout mice at 24 hours after no treatment (basal), LPS (500 ng/ml), αCD200R1 (5 μg/ml) or LPS + αCD200R1. Data represent one experiment and is shown as mean ± standard deviation (SD).
